# Dietary Fiber Is Essential to Maintain Intestinal Size, L-Cell Secretion, and Intestinal Integrity in Mice

**DOI:** 10.3389/fendo.2021.640602

**Published:** 2021-02-26

**Authors:** Jenna Elizabeth Hunt, Bolette Hartmann, Kristina Schoonjans, Jens Juul Holst, Hannelouise Kissow

**Affiliations:** ^1^ Department of Biomedical Sciences, Faculty of Health and Medical Sciences, University of Copenhagen, Copenhagen, Denmark; ^2^ Novo Nordisk Foundation Center for Basic Metabolic Research, Faculty of Health and Medical Sciences, University of Copenhagen, Copenhagen, Denmark; ^3^ Institute of Bioengineering, School of Life Sciences, École Polytechnique Fédérale de Lausanne, Lausanne, Switzerland

**Keywords:** dietary fiber, GLP-2, GLP-1, TGR5 (GPBAR1), L-cell

## Abstract

Dietary fiber has been linked to improved gut health, yet the mechanisms behind this association remain poorly understood. One proposed mechanism is through its influence on the secretion of gut hormones, including glucagon-like peptide-1 (GLP-1) and glucagon-like peptide-2 (GLP-2). We aimed to: 1) investigate the impact of a fiber deficient diet on the intestinal morphological homeostasis; 2) evaluate L-cell secretion; and 3) to ascertain the role of GLP-1, GLP-2 and Takeda G protein-receptor-5 (TGR5) signaling in the response using GLP-1 receptor, GLP-2 receptor and TGR5 knockout mice. Female C57BL/6JRj mice (n = 8) either received a standard chow diet or were switched to a crude fiber-deficient diet for a short (21 days) and long (112 days) study period. Subsequent identical experiments were performed in GLP-1 receptor, GLP-2 receptor and TGR5 knockout mice. The removal of fiber from the diet for 21 days resulted in a decrease in small intestinal weight (p < 0.01) and a corresponding decrease in intestinal crypt depth in the duodenum, jejunum and ileum (p < 0.001, p < 0.05, and p < 0.01, respectively). Additionally, colon weight was decreased (p < 0.01). These changes were associated with a decrease in extractable GLP-1, GLP-2 and PYY in the colon (p < 0.05, p < 0.01, and p < 0.01). However, we could not show that the fiber-dependent size decrease was dependent on GLP-1 receptor, GLP-2 receptor or TGR5 signaling. Intestinal permeability was increased following the removal of fiber for 112 days. In conclusion, our study highlights the importance of dietary fiber to maintain intestinal weight, colonic L-cell secretion and intestinal integrity.

## Introduction

Non-digestible carbohydrates, termed dietary fibers, have been linked to improved health outcome, especially concerning gut health (1). Their presence in the diet can delay gastric emptying rate (2), increase fecal bulk and moisture content (3), and impact bacterial diversity (4) and fermentation (5). Despite these attributes, fiber consumption in the diets of westernized nations continues to fall below the recommended guidelines (6). This ‘western diet’ is characterized by calorie-rich processed foods, high in sucrose and saturated fats with reduced dietary fiber (7) and is linked to the rise in noncommunicable diseases (1) and a decrease in microbial diversity (8, 9). Additionally, low fiber diets are recommended to patients to manage intestinal symptoms such as diarrhea in a range of gastrointestinal conditions including Crohn’s disease, ulcerative colitis, irritable bowel syndrome, as well as following chemotherapy and radiotherapy (10). One proposed mechanism by which diet composition can influence health is through its influence on the secretion of gut hormones, including glucagon-like peptide-1 (GLP-1) and glucagon-like peptide-2 (GLP-2) (11, 12). The glucagon-like peptides are co-secreted from enteroendocrine L-cells, predominantly located in the distal small intestine (SI) and colon. GLP-1 is a multifunctional incretin hormone best known for modulating glucose metabolism (13) but also has a mild trophic effect in the intestine of rodents (14, 15). GLP-2 plays an important role in gut epithelium function by being the major regulator of the intestinal size as well as absorptive capacity (16–19). Together GLP-1 and GLP-2, synergistically ameliorate intestinal injury and improve intestinal healing (20).

Classically, luminal nutrient delivery has been described as the stimulus for enteroendocrine L-cells (21), but more recently other stimuli such as short-chain fatty acids (SCFA) and bile acids have also been reported to be stimulators in experimental animals (22, 23) and humans (24, 25). SCFAs are the primary fermentation products of dietary fiber by bacteria located in the proximal colon (26) and exert their secretagogue function by binding to the free fatty acid receptors 2 and 3 (FFAR2 and FFAR3) located on L-cells (27, 28). The gut microbiota metabolizes and modifies bile acids and regulates the expression of their synthesizing enzymes (29, 30). They exert their secretagogue function by binding to the bile acid receptor Takeda G protein-receptor-5 (TGR5) located on intestinal L-cells (31).

Given the ability of dietary fiber to modulate the gut microbiota (5) and its fermentation products we hypothesized that the removal of dietary fiber would decrease the gut size and available absorptive capacity due to the moderation in L-cell secretion. We aimed to investigate the impact of a fiber deficient diet on the intestinal morphological homeostasis and L-cell secretion and to evaluate GLP-1 receptor (GLP-1r), GLP-2 receptor (GLP-2r) and TGR5 signaling in the response using knockout mice.

## Materials and Methods

### Animals

All experiments adhered to guidelines of Danish legislation governing animal experimentation (1987) and were approved by the Danish animal experiments inspectorate (license no. 2013-15-2934-00833). Female C57BL/6JRj mice, 8 weeks of age, were purchased from Janvier Laboratories, Saint-Berthevin Cedex, France. Female GLP-1r knockout mice (*GLP-1r^-/-^*), 8–12 weeks of age, were generated using the loxP/Cre system as described previously (32). Female GLP-2r knockout mice (*GLP-2r^-/-^*), 8–12 weeks of age, were generated *via* CRISPR/Cas9-mediated gene editing as described previously (33). Female TGR5 knockout mice (*TGR5^-/-^*), 8–12 weeks of age, were bred in-house with permission from the Laboratory of Integrative and Systems Physiology, Ecole Polytechnique de Lausanne, Switzerland (34). All knockout mice were bred by heterozygote breeding and *GLP-1r^+/+^*, *GLP-2r^+/+^* and *TGR5^+/+^* littermates were used as controls. All animals were housed in the same animal facility under standard 12:12 h light-dark cycles with *ad libitum* access to food and water.

### Experimental Setup

#### Fiber-Free Diet in C57BL/6JRj Mice

**Figure 1:**
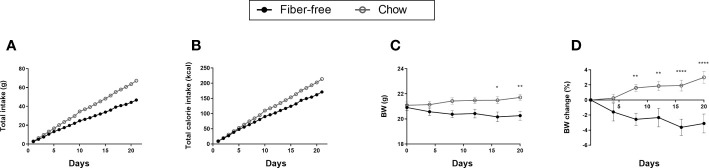
Dietary intake and body weight change in *C57BL/6JRj* mice fed a fiber-free diet for 21 days. **(A)** Total dietary intake calculated by dividing the total food intake (g) per cage by the number of mice in the cage. **(B)** Total calories consumed per mouse. **(C)** Body weight (BW). **(D)** Change in be BW expressed as percentage BW change from day 0. BW and change in BW was compared using a two-way ANOVA followed by a Sidak’s multiple comparisons test and data are presented as mean ± SEM. *p < 0.05, **p < 0.01, and ****p < 0.0001.

Female mice (n = 8), housed 8 per cage, either continued to receive a standard chow diet herein referred to as ‘chow’ (Altromin, Lage, Germany, cat.no. 1310) or were switched to a crude fiber-deficient diet (Altromin, cat.no. 1013) referred herein to as ‘fiber-free’ for 21 or 112 days (**Table 1**; for full nutritional composition, see **Supplementary Data Table 1** https://doi.org/10.6084/m9.figshare.13568261.v1). All animals from the 21 days experiment were weighed every four days and their daily food intake was determined by hand weighing the remaining food per cage. Daily nutritional consumption per mouse was calculated by dividing the total intake (g) per cage by the number of mice in the cage, multiplied by the macronutrient distribution of the diets per g (**Figure 1**).

**Table 1:** Nutritional composition of diets per g/100 g.

Group	Fiber-free	Chow
Fiber	0.17	6.10
Fat	5.08	4.10
Protein	17.61	19.20
Carbohydrate	62.19	40.80
kcal/kg	3670.06	3188.00

Mice were given an intraperitoneal injection of bromodeoxyuridine (BrdU) (50 mg/kg) (Sigma-Aldrich, Missouri, US, cat.no. B5002) (only the 21 days experiment) and an oral gavage of fluorescein isothiocyanate dextran (FITC-dextran) (400 mg/kg) (Sigma-Aldrich, cat.no. 60842-46-8) 2 h before sacrifice. Mice were anesthetized with an intraperitoneal injection of ketamine (90 mg/kg) (MSD Animal Health, Madison, New Jersey, US, cat.no. 511485) and xylazine (10 mg/kg) (Rompun Vet, Bayer Animal Health, Leverkusen, Germany, cat.no. 148999). From a midline incision, a total blood draw was made from the vena cava and the blood was collected in pre-cooled 3.9 mmol/l EDTA-coated tubes (Eppendorf, Hamburg, Germany, cat.no. 20901-757) and stored on ice until centrifuged (3500 rpm, 15 min, 4°C). Plasma was transferred to pre-cooled Eppendorfs and stored at -20°C until further analysis. The small intestine (SI) was resected and partitioned into the duodenum, jejunum and ileum and the colon was resected. All the resected tissue was flushed and weighed as previously described (35). Transverse sections of the duodenum, jejunum, ileum and colon were fixed in 10% neutral formalin buffer for 24 h and subsequently transferred to 70% ethanol until further processing for histology and immunohistochemistry (IHC). Additional tissue samples were collected in the same manner, then snap-frozen and stored at -80°C until protein extraction and radioimmunoassay (RIA) (only 21 days).

#### Fiber-Free Diet in Knockout Mice

Female *GLP-1r^-/-^*, *GLP-2r^-/-^* and *TGR5^-/-^* (n = 8–10) mice and their *GLP-1r^+/+^*, *GLP-2r^+/+^* and *TGR5^+/+^* littermates (n = 8–10) either continued to receive the chow diet (Altromin, cat.no. 1310) or were switched to the fiber-free diet (Altromin, cat.no. 1013) for 21 days. All animals were weighed every four days and intestines were resected, flushed and weighed as described previously.

### Histology

Formalin-fixed tissue from the duodenum, jejunum, ileum and colon was first dehydrated and paraffin-embedded. Slices of the embedded tissue (4 μm) were cut using a microtome (pfm Slide 4005 E, pfm medical, Köln, Germany) and stained with hematoxylin/eosin. The average crypt depth and villus height were approximated by measuring these parameters in 20 well-oriented villi and crypts. Mucosa area was measured by subtracting the luminal circumference from the submucosal circumference. All measurements were made from histological photographs taken using a light microscope connected to a camera (Zeiss Axio Lab.A1, Brock & Michelsen, Birkeroed, Denmark) and Zeiss Zen lite software (Carl Zeiss Microscopy GmbH, Göttingen, Germany) (demonstrated in **Supplementary Data Figure 1A** https://doi.org/10.6084/m9.figshare.13594058.v1). The observer was blinded as to the origin of the section.

### Immunohistochemistry

Slides were stained using the Ultravision Quanto Detection System HRP DAB (Thermo Fisher Scientific, Massachusetts, US, cat.no TL-060-QHD), according to the manufacturers’ instructions and pre-treated by heat-induced epitope retrieval in Tris/EDTA buffer pH 9.0 (Thermo Fisher Scientific, cat.no TA-125-PM4X). Non-specific binding was blocked using Rodent Block Buffer (Ultravision Quanto Mouse on Mouse kit, Thermo Fisher Scientific, cat no. TL-060-QHDM) according to the manufacturers’ instructions. The monoclonal mouse anti-BrdU antibody BU20a (Thermo Fisher Scientific, cat.no MA1-81890, RRID : AB_927209) diluted 1:500 was applied to the tissue for 1 h. Slides were counterstained with hematoxylin and visualized using a light microscope. Proliferation was quantified by counting the total number of crypt cells and calculating the percentage of BrdU positive cells per crypt. At least 20 well-orientated crypts were selected in each section. The observer was blinded as to the origin of the section.

### Protein Extraction

Intestinal tissue was subject to peptide extraction as previously described (33). In short, the tissue samples were homogenized in 1% trifluoroacetic acid (TFA; Thermo Fisher Scientific, cat.no. TS-28904) then left to stand at room temperature for 1 h and were then centrifuged for 10 min at 2000xg. After determination of the concentration of protein (Pierce BCA Protein Assay Kit, Thermo Fisher Scientific, cat.no 23225), the supernatants were fractionated using tc18 cartridges (Waters, Massachusetts, US, cat.no 036810). After evaporation, ethanol-eluted peptides were reconstituted in assay buffer (phosphate buffer 80 mM, 0.1% human serum albumin, EDTA 10 mM, pH 7.5, plus 0.01 mM of the dipeptidyl peptidase-4 inhibitor valine-pyrrolidide).

### Radioimmunoassay

Total concentrations of amidated GLP-1 were quantified using antiserum (code name 89390) targeting the C-terminus of the GLP-1 molecule and reflects the sum of intact GLP-1 (7–36)amide, the primary metabolite GLP-1 (9–36)amide and any other GLP-1(x-36)amide isoforms (36). Intact GLP-2 (1–33) was measured using antiserum (code name 92160) targeting the N-terminus of the GLP-2 molecule (37). Total PYY, sum of 1–36 and 3–36 forms, was quantified using antiserum (code name T-4093) (38). Concentrations were normalized to measured protein content in the extract.

### Plasma Determination of FITC-Dextran

Total intestinal permeability was indirectly measured by the determination of the non-digestible 4-kDa dextran conjugated with fluorescein isothiocyanate (FITC-dextran) in plasma (39). Following oral administration, FITC-dextran transits through the gastrointestinal tract and can passively pass through the intestinal epithelium. The concentration of FITC-dextran in plasma represents the permeability of the intestinal epithelium. Plasma was diluted in an equal volume of phosphate-buffered saline (PBS) and subjected to fluorescence analysis using an excitation wavelength of 485 nm and an emission wavelength of 528 nm in a SpectraMax iD3 multi-mode microplate reader (Molecular Devices, San Jose, US). The results of the fluorescence measurements were compared to a standard curve of known FITC-dextran concentrations.

### Calculation and Statistical Evaluation

All statistics were performed using GraphPad Prism 6 (La Jolla, California, US). Statistical evaluations of the data were carried out using two-sided, unpaired t tests when comparing two independent groups and a two-way analysis of variance (ANOVA) when comparing multiple independent groups followed by a Sidak’s multiple comparisons test. Values of p < 0.05 were considered significant and all data in the text and graphs were presented as mean ± SEM.

## Results

### Fiber-Free Diet in C57BL/6JRj Mice

#### Dietary Intake and Body Weight

After the 21 days of feeding, fiber-free fed mice had a lower total intake of diet per mouse (47 vs. 67 g) (**Figure 1A**). This resulted in a lower total calorie intake (171 vs. 214 kcal per mouse) (**Figure 1B**). Unsurprisingly, total fiber intake after 21 days was lower in the fiber-free group (0.08 vs. 4.11 g). Total fat (2.37 vs. 2.75 g) and carbohydrate (29 vs. 27 g) consumption after 21 days was similar in both treatment groups. Fiber-free mice had a lower total protein intake (8 vs. 13 g) compared to the chow fed mice after 21 days. Body weight (BW) was significantly decreased from day 17 in the fiber-free mice (**Figure 1C**) and the percentage BW change was significantly different from day 8 until the end of the study (**Figure 1D**).

#### Weight and Morphometric Estimates in the Intestine

**Figure 2:**
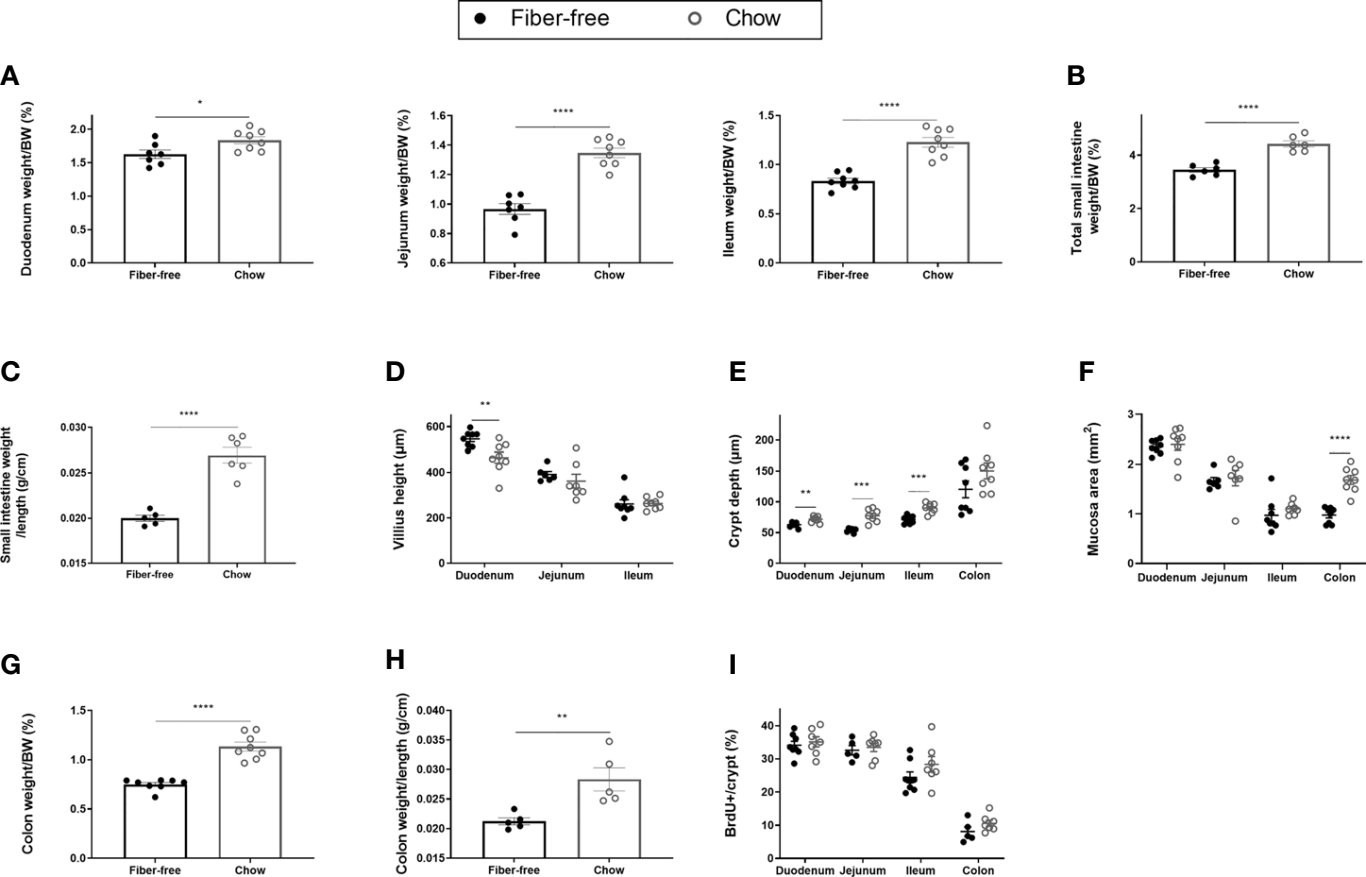
Morphometric estimates in the intestine of *C57BL/6JRj* mice fed a fiber-free diet for 21 days. Weight of the intestine in mice receiving either a fiber-free or chow diet for 21 days. **(A)** Duodenum, jejunum and ileum weight relative to final body weight (BW); **(B)** Total small intestinal (SI) weight relative to BW; **(C)** SI weight relative to SI length; **(D)** SI Villus height; **(E)** SI and colon crypt depth; **(F)** SI and colon mucosa area; **(G)** Colon weight relative to BW; **(H)** Colon weight relative to colon length; **(I)** BrdU immunopositive (BrdU+) cell number expressed as a percentage of total crypt cell number. Data were compared using a two-sided, unpaired students t-test and presented as mean ± SEM. *p < 0.05, **p < 0.01, ***p < 0.001, ****p < 0.0001.

Fiber-free fed mice had significantly reduced intestinal weights, relative to BW, in the duodenum (11%), the jejunum (28%) and the ileum (32%) after 21 days of feeding (**Figure 2A**). Total small intestine (SI) weight relative to BW, was significantly reduced by 25% (**Figure 2B**). Additionally, fiber-free feeding significantly decreased SI weight per length (**Figure 2C**). Given this, surprisingly fiber-free mice had a significant increase in duodenal villus height (**Figure 2D**). Villus height in the jejunum and ileum remained unchanged (**Figure 2D**). Fiber-free feeding significantly reduced crypt depth in the duodenum by 13%, in the jejunum by 36%, and in the ileum by 23%, crypt depth remained unchanged in the colon but the overall mucosa area in the colon was significantly decreased (**Figures 2E, F**). Images of representative hematoxylin and eosin-stained intestinal tissue are displayed in **Supplementary Figures 1B, C**; https://doi.org/10.6084/m9.figshare.13594058.v1). Colon weight, relative to BW, was significantly decreased by 41% (**Figure 2G**) and colon weight per length was significantly decreased (**Figure 2H**). Fiber-free feeding did not affect the number of BrdU immunopositive cells per crypt in the SI or colon (**Figure 2I**).

**Figure 3:**
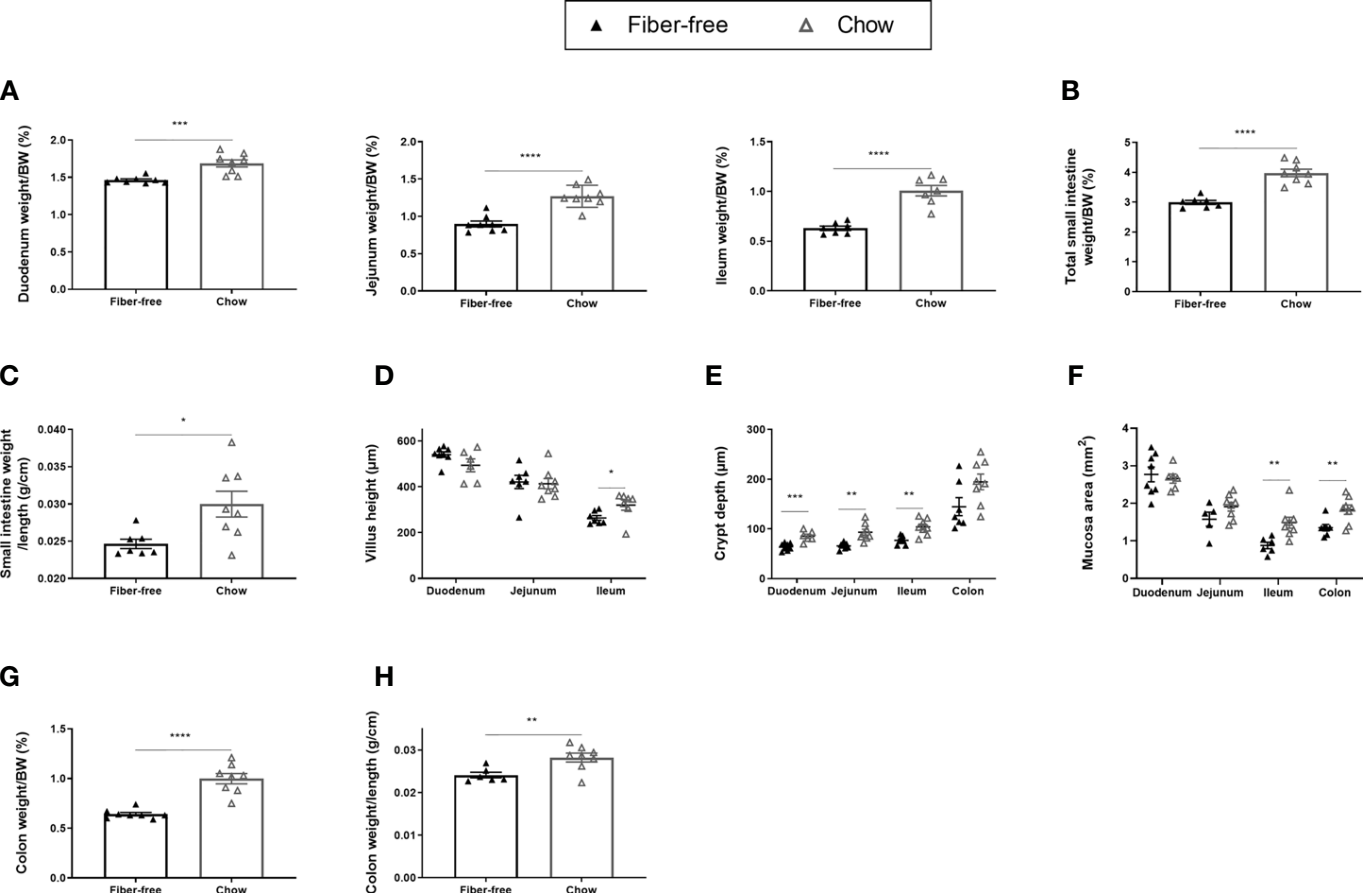
Body weight and morphometric estimates in the intestine of *C57BL/6JRj* mice fed a fiber-free diet for 112 days. Weight of the intestine in mice receiving either a fiber-free or chow diet for 112 days. **(A)** Duodenum, jejunum and ileum weight relative to final body weight (BW); **(B)** Total small intestinal (SI) weight relative to BW. **(C)** SI weight relative to SI length; **(D)** SI Villus height; **(E)** SI and colon crypt depth; **(F)** SI and colon mucosa area. **(G)** Colon weight relative to BW; **(H)** Colon weight relative to colon length. Data were compared using a two-sided, unpaired students t-test and presented as mean ± SEM. *p < 0.05, **p < 0.01, ***p < 0.001, and ****p < 0.0001.

Long-term fiber-free fed mice had significantly reduced intestinal weights, relative to BW, in the duodenum (13%), the jejunum (29%) and the ileum (38%) (**Figure 3A**). Total SI weight, relative to BW, was significantly reduced by 28% (**Figure 3B**) and there was a tendency (p=0.0594) for reduced SI weight per length (**Figure 3C**). Fiber-free feeding did not affect the villus height in the duodenum and jejunum, but in the ileum, the villus height was significantly decreased by 19% (**Figure 3D**). Fiber-free feeding significantly reduced crypt depth in the duodenum by 28%, in the jejunum by 35% and in the ileum by 30% and tended to reduce crypt depth in the colon (p=0.0558) (**Figure 3E**). Mucosa area was significantly decreased in the ileum and colon (**Figure 3F**). Images of representative hematoxylin and eosin-stained intestinal tissue are displayed in **Supplementary Figure 1, panel D and E** https://doi.org/10.6084/m9.figshare.13594058.v1). Colon weight, relative to BW, was significantly decreased by 44% following a long-term fiber-free diet (**Figure 3F**). Colon weight per length was significantly decreased in fiber-free mice compared to chow (**Figure 3G**).

#### L-Cell Hormone Production

**Figure 4:**
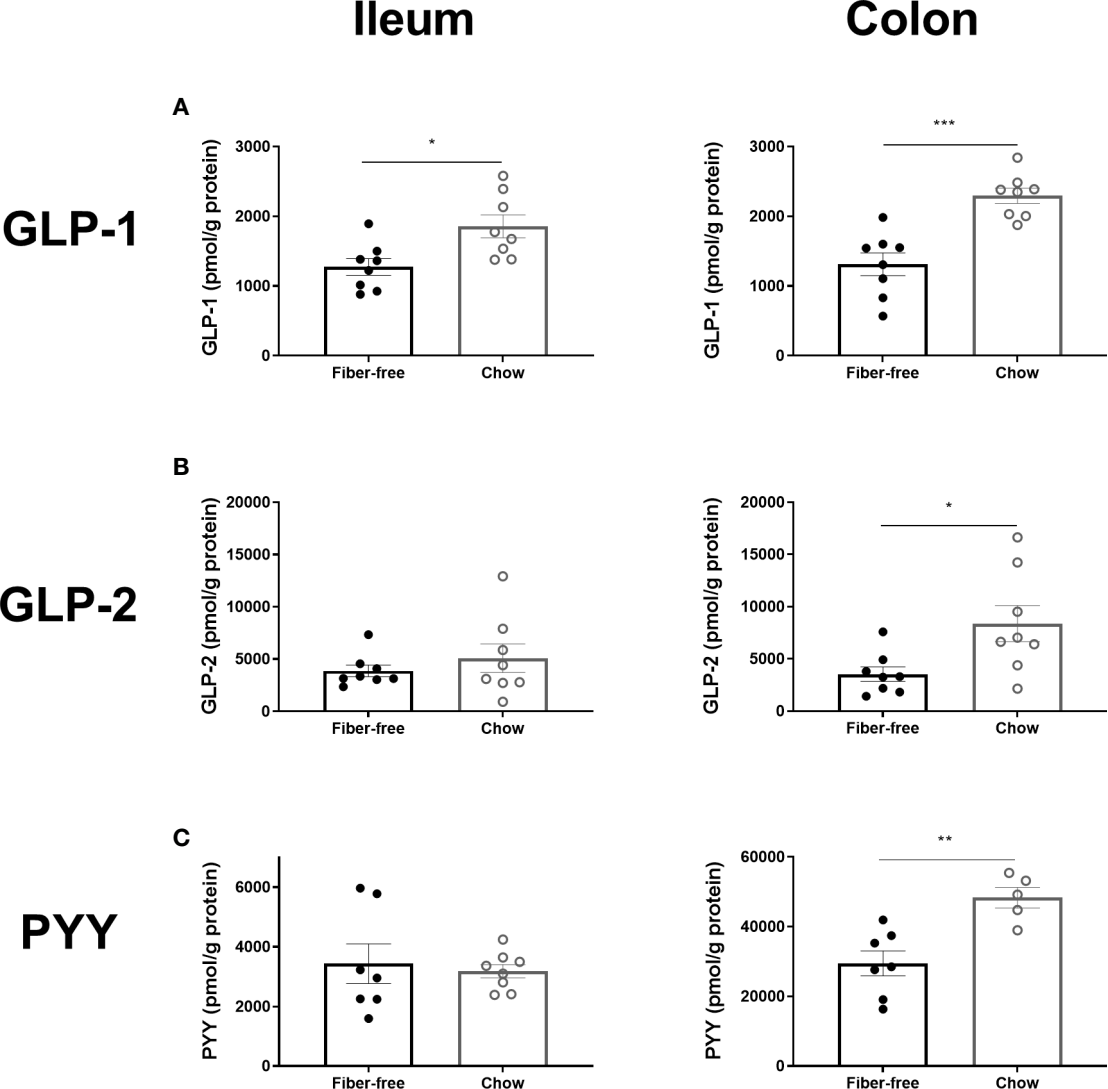
L-cell hormone production in *C57BL/6JRj* mice fed a fiber-free diet for 21 days. L-cell hormone production in mice receiving either a fiber-free or chow diet for 21 days. **(A)** Total amidated GLP-1 (sum of intact GLP-1 (7–36)amide, the primary metabolite GLP-1 (9–36)amide and other GLP-1(x-36) amide isoforms) concentration, normalized to pmol per g protein, in the ileum and colonic tissue. **(B)** Intact GLP-2 (1–33) concentration normalized to pmol per g protein, in the ileum and colonic tissue. **(C)** Total PYY, sum of 1-36 and 3-36 isoforms, concentration, normalized to pmol per g protein, in the ileum and colonic tissue. Data were compared using a two-sided, unpaired students t-test and presented as mean ± SEM. *p < 0.05, **p < 0.01, ***p < 0.001.

Fiber-free feeding for 21 days significantly decreased the concentration of total GLP-1, by 37% and 55% in the ileum and colon, respectively (**Figure 4A**). The concentration of intact GLP-2 (1–33) remained unchanged in the ileum but was significantly decreased in the colon by 80% (**Figure 4B**). Total PYY, remained unchanged in the ileum but was significantly decreased by 48% in the colon (**Figure 4C**).

#### Intestinal Permeability

**Figure 5:**
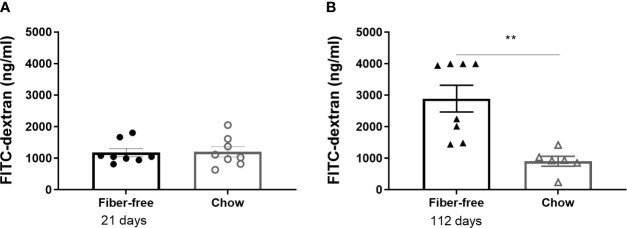
Intestinal permeability in *C57BL/6JRj* mice fed a fiber-free diet for 21 and 112 days. Intestinal permeability in mice receiving either a fiber-free or chow diet **(A)** for 21 or **(B)** 112 days measured as the level of FITC-dextran in sera 2 h post oral administration. Data were compared using a two-sided, unpaired students t-test and presented as mean ± SEM. **p < 0.01.

Fiber-free feeding did not affect the level of FITC-dextran in the plasma after 21 days (**Figure 5A**). After 112 days, the concentration of plasma FITC was significantly tripled in the fiber-free mice compared to chow (**Figure 5B**).

### Fiber-Free Diet in Knockout Mice

**Figure 6:**
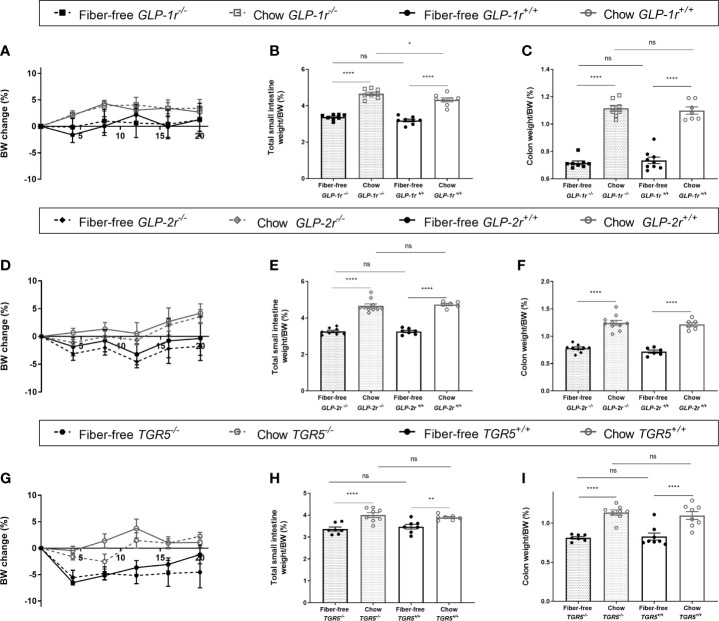
Fiber-free diet in in knockout mice. *GLP-1r^-/-^*, *GLP-2r^-/-^* and *TGR5^-/-^* mice and their *GLP-1r ^+/+^*, *GLP-2r^+/+^* and *TGR5^+/+^* littermates received either a fiber-free or chow diet for 21 days. **(A, D, G)** Body weight (BW) change, expressed as percentage BW change; **(B, E, H)** Small intestinal (SI) weight, normalized to BW; **(C, F, I)** Colon weight normalized to BW. Data were compared using a two-way ANOVA followed by a Sidak’s multiple comparisons test and data are presented as mean ± SEM. **p < 0.01, ****p < 0.0001 and ns, non-significant.

In *GLP-1r^-/-^* and *GLP-1r^+/+^* mice, genotype did not affect BW change (**Figure 6A**). Total SI weight, normalized to BW, was significantly increased in the chow fed *GLP-1r^-/-^* mice compared to chow *GLP-1r^+/+^* with no differences between genotypes found in the fiber-free mice (**Figure 6B**). Genotype did not affect colon weight normalized to BW (**Figure 6C**). In *GLP-2r^-/-^* and *GLP-2r^+/+^* mice, genotype did not affect BW change, SI or colon weight normalized to BW (**Figures 6D–F**). Similarly, in the *TGR5^-/-^* and *TGR5^+/+^* mice, genotype did not affect BW change, SI or colon weight normalized to BW (**Figures 6G–I**).

## Discussion

This study emphasizes the importance of dietary fiber for maintaining intestinal weight, colonic L-cell secretion and intestinal integrity in mice. We show that the removal of crude fiber from the diet dramatically decreased the intestinal size during both the short (21 days) and long-term (112 days) study periods and, additionally, drastically decreased colonic L-cell hormone content after 21 days. Intestinal permeability was unaffected following the 21 days deficient fiber feeding but was significantly increased after 112 days of feeding.

Traditionally, the physiological influence of dietary fiber was thought to be limited to the intestinal lumen, affecting gastric emptying rate (2), contributing to fecal bulk and moisture content (40). These attributes made fiber an ideal candidate to maintain healthy intestinal transit, yet increasingly fiber and its derivatives have been shown to play a role beyond lumen. Fiber has been shown to alter the physical characteristics of the rodent gut, such as increasing intestinal weight (41) and increasing intestinal epithelial cell proliferation (42), which could make fiber a potential candidate for ameliorating intestinal injury. These effects could also have implications for populations consuming low fiber diets such as part of a western diet or for patients following advice to consume low-fiber diets to alleviate intestinal side effects such as diarrhea (10).

To assess the impact of low fiber diets on the gut size, we switched mice from their normal chow diet to a fiber-deficient diet for 21 days. Here, we show that removal of crude dietary fiber significantly decreased the weight of the SI and, to a larger extent, the colon. Additionally, removal of fiber significantly decreased crypt depth in the SI and significantly decreased the mucosa area in the colon. These findings support previous data describing a dose-dependent effect of fiber on gut size in rats (41). Similar to our study, the authors reported a fiber-mediated effect on crypt depth in the jejunum and ileum. Additionally, they report that fiber supplementation increased villus height whereas paradoxically we report a significant increase in villus height, in the duodenum, upon the removal of fiber after 21 days. This difference in outcome could be attributed to the different experimental setup, with Adam et al. (41) supplementing diets with the soluble fiber pectin, compared to the total removal of crude fiber exemplified in this study. The total removal of fiber could trigger a compensatory response in the proximal SI due to a loss of absorptive capacity, as a product of reduced intestinal size.

We further investigated this possible compensatory response by repeating the experiment but with a long-term feeding schedule of 4 months (112 days). Comparable to the 21 days feeding regime, long-term fiber deficiency reduced SI and colon weights by a similar proportion that coincided with a decrease in crypt depth in the small intestine and a decrease in mucosa area in the colon. Uniquely in the long-term study, there was a decrease in villus height and overall mucosa area in the ileum following fiber-free feeding. These results suggest that the fiber-free fed mice were not able to compensate for their loss of intestinal weight over time. Instead, the reduction in crypt depth preceded villous atrophy in the ileum. At the cell level, atrophic loss may be a product of decreased proliferation or increased apoptosis. Here, we report no changes in proliferative activity measured using BrdU immunopositivity, therefore, it could be speculated that the atrophic loss was due to an increased apoptotic rate.

The observed decrease in colonic weight could be assumed to be a product of the decreased fermentation processes, yet similar to previous findings utilizing soluble fiber in rats (41), the morphological changes were also present in the SI. This suggests that the observed growth effect was independent of the local actions of dietary fiber fermentation. Instead, we hypothesized the growth response in the SI could be mediated by the intestinal gut hormones GLP-1 and GLP-2. GLP-2 is a well-documented intestinal tropic hormone shown to increase proliferation and reduce apoptosis (16) (17) (43). GLP-1 can protect against mucosal loss following intestinal injury (44). Both hormones are co-secreted upon a number of nutritional stimuli including in response to dietary fiber fermentation products (22) (27). Indeed, we show that the absence of fiber in the diet decreased the tissue levels of GLP-1, GLP-2 and additionally PYY. The present results agree with previous studies assessing L-cell secretion following fiber supplement in rodents and humans (3) (45) (46). However, in contrast to measuring plasma hormone levels we measured the local tissue concentrations and showed that the L-cell hormones were primarily affected in the colon and to a lesser extent in the distal SI. This ultimately suggests that the fiber-mediated stimulation of the gut growth is controlled by colonic L-cells. Targeting this colonic endocrine function has a large therapeutic potential since the largest density of L-cells is found in the colon (47). These L-cells are situated away from classical luminal nutrient stimulation thereby attributing a different source of stimuli for this subset of L-cells that we are just beginning to characterize.

Given that a decrease in intestinal size corresponded with a decrease in L-cell content in the colon following a fiber deficient diet, we hypothesized that the intestinotrophic actions of GLP-2 and to a lesser extent GLP-1 could drive this response and investigated our hypothesis using global

*GLP-1r^-/-^* and *GLP-2r^-/-^* mice. Abolition of GLP-1 receptor signaling did not affect the decreased SI or colon weight observed following deficient dietary fiber feeding; however, loss of fiber from the diet removed the significant increase in SI weight observed in *GLP-1r^-/-^* mice. This suggests an interaction between GLP-1 signaling, gut size and the presence of fiber, whereby in the presence of fiber, *GLP-1r^-/-^* mice develop increased SI weight, while the signal is lost following the removal of fiber. Abolition of GLP-2 receptor signaling did not affect the intestinal size. To assess if the growth response could be impacted by the indirect manipulation of GLP-1 and GLP-2 we used the bile acid receptor *TGR5^-/-^* mice. TGR5 is located on the L-cell and we have previously shown that TGR5 stimulation led to a GLP-2 mediated increase in intestinal size from colonic L-cells (33). However, in the current experiments intestinal size was not impacted by TGR5 signaling. Therefore, we were not able to show the mechanistic drivers of the fiber-mediated growth response using these knockout mouse models. Future studies should focus on assessing the contribution of other microbial modulated metabolites or by assessing receptor contributions in inducible knockout models since germline knockout models, as used in this study, are limited by the risk of evolved compensatory mechanisms to maintain their intestinal capacity.

Dietary fiber has been proposed to improve gut and overall health by helping maintain the intestinal barrier (4, 48). Upon the removal of dietary fiber, bacteria switch their glycan metabolism from fiber degradation to mucus glycan degradation thereby reducing the colonic mucus layer thickness which increases microbial translocation, triggering systemic inflammation (4, 48, 49). Here, we investigated intestinal permeability following the removal of dietary fiber using FITC-dextran. Surprisingly, there were no differences in permeability after 21 days but a large increase after 112 days of fiber-free feeding. This suggests that long-term fiber interventions are required to moderate permeability to an extent that can be detected at the serum level but does not rule out short-term precursor modifications such as mucus layer thickness, which were not assessed in this study.

The present study is limited by a difference in the composition of the two compared diets. Both diets were purchased from the same distributor but had micro and macronutrient differences beyond the content of fiber including a higher kcal/kg in the fiber-free diet. Likely due to the higher kcal/kg, fiber-free fed mice consumed less diet which at the end of the 21 day feeding period increased the disparities between macronutrient consumption and led to a significant decrease in BW from day 8. In particular, after 21 days the fiber-free mice had consumed less protein compared to the chow mice (8 vs. 13 g) therefore, we cannot exclude the influence of decreased protein in the investigated intestinal parameters. Despite this, previous studies investigating the effect of low protein diets on the intestine have shown they correlate with a significant reduction in villus heights (50, 51) which was not observed in our study. Additionally, protein diets have been shown to have little effect on the GLP-2 secretion (52).

Despite the numerous studies describing the health benefits of dietary fiber consumption in humans, the doses needed to emulate the benefits are often not realized in practice due to a combination of logistical reasons affecting compliance and adverse effects such as bloating (53) and flatulence (54). Therefore, it remains important to investigate the mechanisms behind these health benefits to develop dietary recommendations to yield new approaches to prevent or treat a range of human diseases. Here, we show that fiber is essential to maintain intestinal size, colonic L-cell hormone levels and maintain intestinal integrity. These findings could have important implications for populations consuming low-fiber diets such as part of a western diet, who could have an increased susceptibility to intestinal disease. In particular, patients recommended to consume low-fiber diets following intestinal injury, such as in the case of pelvic radiotherapy (55), could be at particular risk since despite the low-fiber diet improving intestinal side effects such as diarrhea, simultaneously it might prolong the radiation-induced intestinal damage (56). Despite showing here that a deficient fiber status dramatically altered the colonic endocrine function, the mechanistic drivers of this response were not elucidated. The spatiotemporal location of the colonic endocrine cells implicates alternative stimuli to classical luminal nutrients such as microbially modulated metabolites like SCFA that are produced and bile acids that are modified and by bacteria-driven processes. Given this, future fiber mediated metabolomics studies are necessary to define likely candidates of L-cell secretion in the colon.

## Data Availability Statement

The original contributions presented in the study are included in the article/**Supplementary Material**. Further inquiries can be directed to the corresponding author.

## Ethics Statement

The animal studies were reviewed and approved by the Danish animal experiments inspectorate.

## Author Contributions

JEH and HK planned and designed the study. KS provided analytical material. JEH and HK performed the experiments. JEH and BH analyzed the results and JEH and HK interpreted the results of the experiment. JEH drafted the manuscript. JEH, JJH, and HK critically revised and edited the manuscript. All authors contributed to the article and approved the submitted version.

## Funding

This work was supported by the Lundbeck Foundation (Grant No. R263-2017-3740), Dagmar Marshall foundation, Aase og Ejnar Danielsens Fond, Agnes og Poul Friis fond, Læge Sofus Carl Emil Friis og Hustru Olga Doris Friis' Legat and was supported by the Novo Nordisk Foundation Center for Basic Metabolic Research (Novo Nordisk Foundation, Denmark).

## Conflict of Interest

The authors declare that the research was conducted in the absence of any commercial or financial relationships that could be construed as a potential conflict of interest.
