# Supplementary material for: Dietary Fiber Is Essential to Maintain Intestinal Size, L-Cell Secretion, and Intestinal Integrity in Mice
Source: Front Endocrinol (Lausanne). 2021 Feb 26;12:640602. doi: 10.3389/fendo.2021.640602 (PMC7953038; doi:10.3389/fendo.2021.640602)
Supplement: Supplementary file 1 [file Image_1.pdf]

Supplementary figure 1

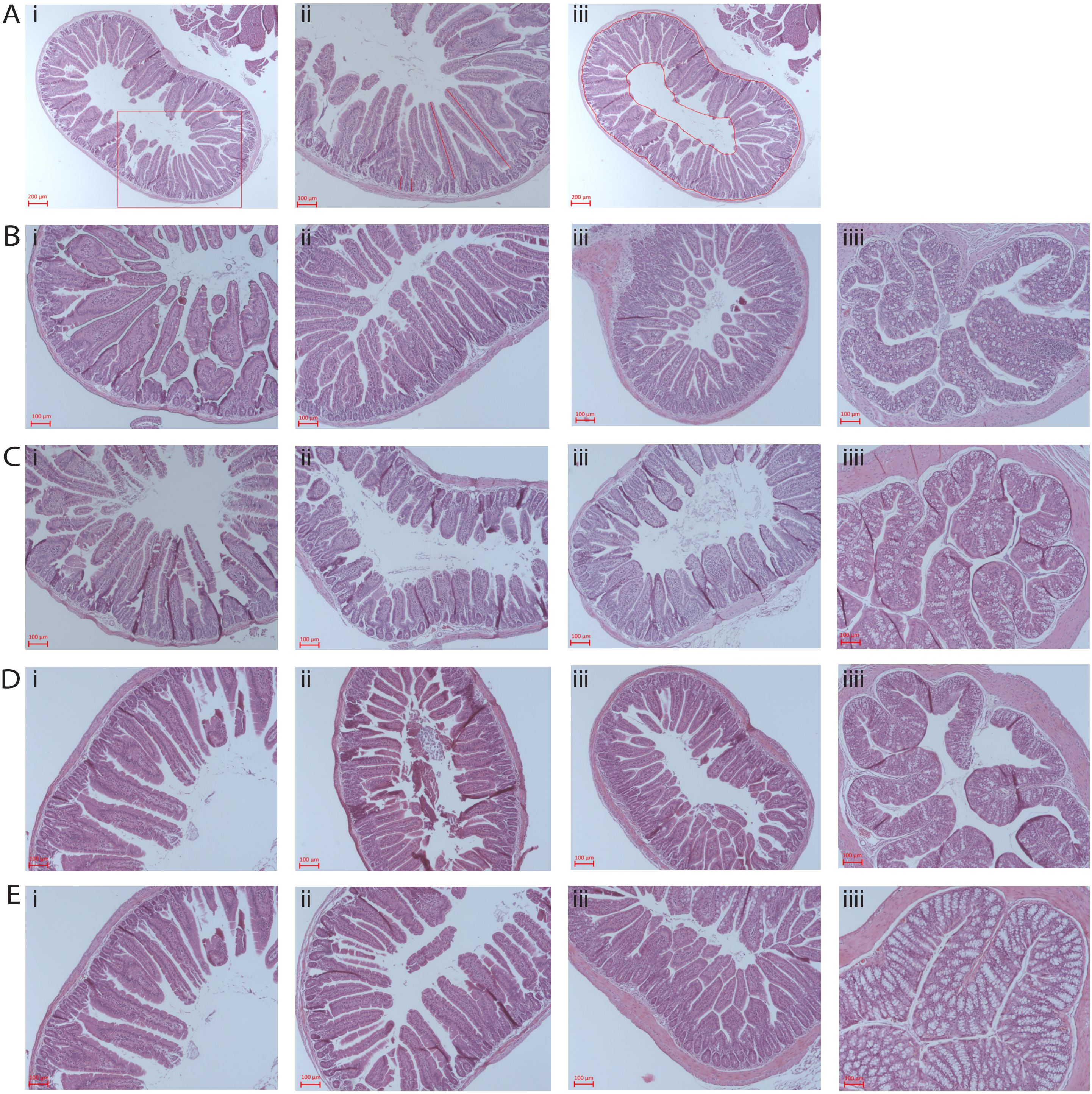

Supplementary figure 1 histological photographs.

Hematoxylin and eosin-stained intestinal tissue. Image **(Ai and ii)** demonstrating villus height and crypt depth measurement. Image **(Aiii)** demonstrating mucosa area measurement. Fiber-free fed mice for 21 days **(Bi)** duodenum, **(Bii)** jejunum, **(Biii)** ileum and **(Biiii)** colon. Chow fed mice for 21 days **(Ci)** duodenum, **(Cii)** jejunum, **(Ciii)** ileum and **(Ciiii)** colon. Fiber-free fed mice for 112 days **(Di)** duodenum, **(Dii)** jejunum, **(Diii)** ileum and **(Diiii)** colon. Chow fed mice for 112 days **(Ei)** duodenum, **(Eii)** jejunum, **(Eiii)** ileum and **(Eiiii)** colon.
