# Supplementary material for: Dietary Fiber Is Essential to Maintain Intestinal Size, L-Cell Secretion, and Intestinal Integrity in Mice
Source: Front Endocrinol (Lausanne). 2021 Feb 26;12:640602. doi: 10.3389/fendo.2021.640602 (PMC7953038; doi:10.3389/fendo.2021.640602)
Supplement: Supplementary file 2 [file Table_1.docx]

**Supplementary data**

Table 1. Nutritional comparison of *fiber-free* diet and *Chow*

|  |  | C1013 *Fiber-free* | 1310 *Chow* |
| --- | --- | --- | --- |
| Metabolized energy | Fat | 457.0 | 463.0 |
| (kcal/kg) | Protein | 691.0 | 901.0 |
|  | Carbohydrates | 514.0 | 1976.0 |
|  |  |  |  |
|  |  |  |  |
| Crude nutrients and moisture | Moisture | 81100.0 | 111241.0 |
| (mg/kg) | Crude Ash | 54870.0 | 61128.0 |
|  | **Crude Fibre** | **1650.0** | **45480.0** |
|  | Crude Fat | 50830.0 | 51398.0 |
|  | Crude Protein | 172650.0 | 225155.0 |
|  | Nitrogenfree extractives | 638900.0 | 505599.0 |
|  |  |  |  |
|  |  |  |  |
| Carbohydrates | Monosaccharides | 0.0 | 0.0 |
| (mg/kg) | Disaccharides | 137305.0 | 54151.0 |
|  | Polysaccharides | 471727.0 | 350300.0 |
|  |  |  |  |
|  |  |  |  |
| Minerals | Calcium | 9308.0 | 7062.0 |
| (mg/kg) | Potassium | 7092.0 | 10144.0 |
|  | Magnesium 2,055 | 685.0 | 2055.0 |
|  | Sodium 2,154 | 2486.0 | 2154.0 |
|  | Phosphorus | 7529.0 | 5090.0 |
|  |  |  |  |
| Trace elements | Aluminium | 2.8 | 81.9 |
| (mg/kg) | Chlorine | 3630.0 | 3382.9 |
|  | Iron | 178.5 | 191.0 |
|  | Flourine | 4.2 | 3.1 |
|  | Iodine | 0.5 | 1.5 |
|  | Cobalt | 0.2 | 0.4 |
|  | Copper | 5.8 | 13.9 |
|  | Manganese | 100.8 | 77.7 |
|  | Molybdenum | 0.2 | 1.5 |
|  | Sulfur | 2791.5 | 974.4 |
|  | Selenium | 0.3 | 0.3 |
|  | Zinc | 29.2 | 85.0 |
|  |  |  |  |
| Amino acids | Alanine | 2528.0 | 10284.0 |
| (mg/kg) | Arginine | 9829.0 | 14822.0 |
|  | Aspartic acid | 3583.0 | 21735.0 |
|  | Cystine | 3196.0 | 3244.0 |
|  | Glutaminc acid | 23675.0 | 43649.0 |
|  | Glycine | 3136.0 | 9565.0 |
|  | Histidine | 5276.0 | 5508.0 |
|  | Isoleucine | 7223.0 | 9668.0 |
|  | Leucine | 14763.0 | 17123.0 |
|  | Lysine | 17401.0 | 11326.0 |
|  | Methionine | 7223.0 | 3171.0 |
|  | Phenylalanine | 7172.0 | 10549.0 |
|  | Proline | 12763.0 | 0.0 |
|  | Serine | 5268.0 | 0.0 |
|  | Threonine | 7154.0 | 0.0 |
|  | Tryptophan | 1977.0 | 0.0 |
|  | Tyrosine | 9285.0 | 0.0 |
|  | Valine | 3296.0 | 0.0 |
|  |  |  |  |
| Fatty Acids | Arachidic acid C-20:0 | 50.0 | 148.0 |
| (mg/kg) | Eicosanoic acid C-20:1 | 150.0 | 185.0 |
|  | Alpha-Linolenic acid C-18:3 | 150.0 | 3018.0 |
|  | Linolenic acid C-18:2 | 28500.0 | 21996.0 |
|  | Palmitic acid C-16:0 | 2500.0 | 5342.0 |
|  | Stearic acid C-18:0 | 1350.0 | 1615.0 |
|  | Oleic acid C-18:1 | 13500.0 | 9287.0 |
|  |  |  |  |
| Added vitamins |  |  |  |
| (IU/kg) | Vitamin A | 15000.0 | 41250.0 |
| (IU/kg) | Vitamin D3 | 500.0 | 1650.0 |
| (mg/kg) | Vitamin E | 180.0 | 210.0 |
|  | Vitamin K3 | 10.0 | 8.0 |
|  | Vitamin B1 | 20.0 | 50.0 |
|  | Vitamin B2 | 20.0 | 33.0 |
|  | Vitamin B6 | 15.0 | 25.0 |
| (µg/kg) | Vitamin B12 | 41.0 | 66.0 |
| (mg/kg) | Nicotinic acid | 50.0 | 99.0 |
|  | Pantothenic acid | 50.0 | 58.0 |
|  | Folic acid | 10.0 | 6.0 |
| (µg/kg) | Biotin | 201.0 | 555.0 |
| (mg/kg) | Choline chloride | 1012.0 | 1650.0 |
|  | Vitamin C | 20.0 | 99.0 |
